# Well-Being at Work after Return to Work (RTW): A Systematic Review

**DOI:** 10.3390/ijerph17207490

**Published:** 2020-10-15

**Authors:** José-María Figueredo, Cristina García-Ael, Andrea Gragnano, Gabriela Topa

**Affiliations:** 1 International School of Doctorate, National Distance Education University (UNED), 28040 Madrid, Spain; jfiguered4@alumno.uned.es; 2 Department of Social and Organizational Psychology, National Distance Education University (UNED), 28040 Madrid, Spain; cgarciaael@psi.uned.es; 3 Department of Psychology, University of Milano-Bicocca, 20126 Milan, Italy; andrea.gragnano@unimib.it

**Keywords:** return to work, work–health balance, subjective well-being, psychological well-being at work, work–health incompatibility, job satisfaction

## Abstract

Background: Employees’ well-being at work after the return to work (RTW) is considered a key aspect of rehabilitation and maintenance of workability. This systematic review aimed at identifying the common psychosocial factors that predict the subjective and psychological well-being in RTW processes after having a long-standing health problem or disability. Objective: To evaluate the subjective and psychological well-being at work of employees with chronic or long-standing health problems or those returning to work after any cause of disability. Data source: Systematic review of articles published in English or Spanish using PsycINFO, PsycARTICLES, MEDLINE, Psychology, and Behavioral Sciences Collection, and Pubpsych. An additional study was identified by contacting expert academics in the field. The search equations used included terms such as Return to Work, Long-Standing Health Problems or Disability, Work Health Balance, and job satisfaction or subjective well-being. Eligibility criteria for the studies: Studies that included a measure of employees’ well-being at work following return to work were selected for the review. Evaluation of the studies and synthesis methods: The studies were selected using predefined fields which included quality criteria. Results: Of the 264 articles returned by the initial search, a total of 20 were finally selected. Results were organized around the three different theoretical approaches for understanding RTW and its antecedents and consequences: (a) RTW and autonomy at work have a positive effect on psychological well-being; (b) job demand is linked to less job satisfaction, whereas a higher level on the work–health balance is associated with job satisfaction and work engagement; (c) internal and external support is linked to job satisfaction in the case of a disease. Limitations: The evidence provided by the results is restricted by the limited availability of studies focusing on well-being at work following return to work. Moreover, the studies identified are of different kinds, thereby preventing comparisons. Conclusions and implications of the main findings: Employees’ subjective well-being after return to work has received very little attention to date. Given its importance in the current configuration of the labor market, it should be the object of more research.

## 1. Introduction

### 1.1. Justification

**Figure 1:**
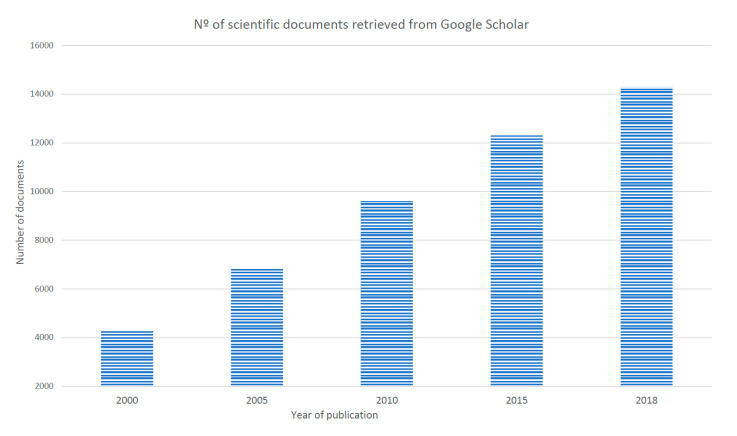
Number of scientific documents retrieved with RTW as keyword.

Return to work (RTW) is described as a multidimensional process influenced by psychosocial factors rather than by medical factors [1,2]. The number of scientific documents that could be retrieved from Google Scholar significantly increases from 2000 to 2020, as Figure 1 shows. However, although a large number of studies (and reviews) have been conducted to develop a greater understanding of factors influencing the RTW concerning specific diseases (e.g., cancers, common mental disorder) and non-diseases (maternity), some of them are segmented based on the diagnosis generating the work leave (e.g., musculoskeletal disorders, cancers, and common mental disorders, maternity), except a few cases [3,4], while others are more oriented to determining barriers and facilitators for work participation than the participants’ well-being as an outcome [5], or mainly focused on the assessment of RTW interventions [6]. Nevertheless, these studies have identified a vast number of work-related psychosocial factors such as level of strain (job demands), self-efficacy, perceived work ability, anxiety, and depression [7], which are (potentially) predictors of RTW and can affect well-being [4].

In general, research into well-being is a broad domain [8] split into two research areas: Subjective or general well-being (a state of complete physical, mental and social well-being) [9] and psychological or work-related well-being (the overall quality of an employee’s experience and functioning at work) [10]. Despite this, RTW research is confusing and fails to give a clear overview of the well-being. That is, some studies explain which vein of research is measured (psychological and/or subjective well-being), whereas the remaining measure job and/or life satisfaction without clearly specifying what these variables refer to.

Therefore, the main objective of this systematic review is to analyze what factors influence subjective and psychological well-being in the RTW processes. For this purpose, this study will be based on a cross-disease approach focused on similarities rather than on the specificity of the different diseases. Due to the fact that the diagnosis of a serious health problem during early adulthood could constitute a personal transition [11], it seems that illness could constitute a key moment for professional development which could affect both psychological and work-related well-being.

### 1.2. The Working Population with Long-Standing Health Problems or Disability

In 2015, the prevalence of long-standing health problems or disability (LSHPD) among the working population of the European Union ranged between 12.8% (16–24 years) and 36.3% (55–64 years) [12]. Moreover, these figures are rising. In 2019, 40.7% of the working population of the European Union aged between 55 and 64 reported some kind of LSHPD, and in most developed countries throughout the world, the percentage of workers with a health problem or disability has increased considerably [13].

The rise in retirement age [14], early diagnosis, and better medical treatment for serious diseases have all contributed to increasing patients’ survival rate and quality of life [15]. Moreover, the greater incidence of chronic health problems due to unhealthy lifestyles [16] has also increased the number of employees with LSHPD.

For example, international reports establish the mean return-to-work rate among cancer patients at 62.5% [17,18]. In the case of cardiovascular diseases, therapeutic procedures have helped reduce mortality rates [19,20], with the consequence being a much greater presence of heart disease among the labor force [21].

It is worth mentioning that cancer survivors and those with other chronic diseases (e.g., stroke, diabetes, heart disease, arthritis, etc.) have similar residual occupational disability levels, which are nevertheless much higher than those of their same-age counterparts with no reported chronic disease [22]. Thus, the majority of patients who have recovered from a disease may continue to experience ongoing negative consequences of the illness itself or the treatment (including pain, fatigue, and low mood), which may, in turn, affect their daily functioning, including work-related aspects [23].

### 1.3. Return to Work and Work Retention

In light of the above, return to work after the sick leave has gradually attracted an increasing amount of attention in the international literature [24,25,26,27,28,29,30,31,32,33,34,35,36]. There is currently great interest in understanding the work environment resources which facilitate or hinder the success of these professional transitions since they represent potentially challenging periods in workers’ careers.

Those who return to work have to reactivate work-related skills in order to readjust to the demands of both their job and/or health-related situation. Within this field, studies have been conducted on the social costs of absence (for example, [37,38]), how to improve the quality of life of those returning to work (for example, [37,39]), and factors which predict a successful RTW (see the systematic review by [4]). To date, these have been the key issues on which research in the field has focused, and much less is known about other factors that are essential for ensuring that returning workers stay in their jobs, preventing absenteeism, and fostering successful, sustainable, and long-term RTW [40].

Returning to work after an illness (and staying there) is associated with better physical and psychological functioning. It has been shown that not working is linked to lower self-esteem, less self-efficacy, and a weaker belief in one’s ability to return to the workplace [41]. Having a job is important, not just for individual and socioeconomic reasons [42], but also because it is believed that being out of work causes, contributes to, and exacerbates adverse health outcomes [43,44]. Moreover, work is an important component of quality of life [45]. There are many benefits, including improved mental and physical health and better social support and financial resources [46]. Despite all this, however, very few studies have explored employees’ adjustment to their psychosocial work environment following sickness absence.

It should be noted that following these life events, workers’ priorities often change. For example, stroke survivors have reported a change in priorities following their illness, attaching less importance to work, and focusing more on improving their health and well-being [47]. This may also be the case among survivors of other serious diseases or non-diseases [48] that require certain lifestyle changes in order to ensure better health and quality of life.

### 1.4. Well-Being at Work after Return to Work

Although research into well-being is a broad domain that has flourished in recent decades [49,50], RTW research is sometimes somewhat confusing in explaining this construct. Research on well-being is organized into two broad traditions: subjective well-being (hedonic well-being), and psychological well-being (eudaimonic well-being) [8]. Subjective well-being can be defined as a positive evaluation of one’s life associated with good feelings measured by life satisfaction, self-esteem, and happiness (balance between positive and negative affect). Psychological well-being is more than just a sense of happiness and satisfaction with life [51]. Generally, psychological well-being or work-related well-being is defined as the overall quality of an employee’s experience and functioning at work [8]. Psychological well-being has been operationalized along three dimensions covering satisfaction–dissatisfaction, enthusiasm–depression, and comfort–anxiety [10]. The former is typically represented by job satisfaction. The second dimension (physical well-being) refers to physiological indicators of health or illness in the workplace. The third facet covers social well-being (e.g., interpersonal relations, levels of social support, the fairness of treatment) [10]. In general, studies on work-related well-being have been focalized on one of the three dimensions. The first approach highlights the features of job content and social context. The second line of research proposes that well-being as a function of balancing the demands of the work and resources available. The third source of research focuses on the quality of working life [8].

There are a lot of studies on the three different approaches, showing that psychological well-being at work is an outstanding key indicator of organizational behavior. For example, it has been demonstrated that job satisfaction has a positive correlation with job performance and a negative association with employee turnover rates [52,53,54,55,56,57]. Nevertheless, little is known about the subjective and psychological well-being of workers with LSHPD after RTW. Both of them may be key in RTW. Research into RTW after overcoming the critical phase of an illness or disability seems still limited. This review adopts a cross-disease framework aimed at identifying the degree of (subjective and psychological) well-being at work among those returning to their jobs after the acute phase of an illness. To the best of our knowledge, very few studies consider these aspects, and no systematic review has previously been carried out in this specific field.

### 1.5. Aims

We aimed to identify those studies which, in one way or another, assess employees’ subjective (i.e., life satisfaction, self-esteem, happiness) and psychological (i.e., job satisfaction, work engagement) well-being at work following their RTW. Since the RTW research is sometimes unclear regarding the definition and measurement of this construct [47], we included all studies which assessed employees’ subjective and psychological well-being following their RTW, regardless of the measurement instrument or procedure used, for the following reasons:

Although the well-being traditions have evolved separately, increasing evidence concludes that psychological and subjective well-being are related but distinct aspects of positive (negative) psychological functioning [49]. In this vein, several studies have found moderate associations between self-efficacy and environmental mastery (two psychological well-being scales) and dimensions of happiness and life satisfaction, although the remaining four dimensions of psychological well-being (i.e., autonomy, positive relations with others) showed weak relationships to subjective well-being [49]. Secondly, the majority of studies focused on this issue have analyzed a broad variety of measures without specifying what type of well-being is being measured.

This systematic review aims to answer the following questions: (a) Are there any studies that assess the subjective and psychological well-being at work of employees with LSHPD after their return to work? (b) What constructs and instruments were used in each case to carry out the measurements? (c) What results were obtained?

## 2. Methods

### 2.1. Protocol

The methodology and results of the review are presented following the standards established in the Preferred Reporting Items for Systematic Reviews and Meta Analyses (PRISMA) guide [58]. Similarly, the detailed explanations provided about this guide [59] were also taken into account, along with the contributions made by [60].

### 2.2. Eligibility Criteria

The bibliographic search aimed to identify all published articles assessing employees’ well-being at work or job satisfaction after their RTW. All articles focusing on people with LSHPD were included.

The search was limited to articles written in either Spanish or English, published between the year 2000 and the moment at which the search was run (21 January 2019). No other constraints were applied in the search fields. This was done to ensure the highest number of hits, given that the topic being studied has, to date, received very little attention from researchers.

### 2.3. Data Sources

The articles were identified through an extensive search in electronic databases. Following this, the bibliography of the returned articles was examined and academics specializing in RTW were consulted (Andrea Gragnano, University of Milan, Milan, Italy). Searches were conducted in the following databases: PsycINFO, PsycARTICLES, MEDLINE, Psychology, and Behavioral Sciences Collection and Pubpsych, CAIRN, Dialnet, PROQUEST Research Library. The final bibliographic search was carried out on 20 August 2019.

### 2.4. Search

Different synonymous or equivalent terms linked to well-being at work were used in combination with those associated with return to work (RTW). The search equations used were as follows:

“Job satisfaction” OR “Well-Being” AND “Return to work” OR “RTW”

“Subjective well-being” OR “Life Satisfaction” AND “return to work” OR “RTW”

“Job satisfaction” OR “Well-Being” OR “Return to work” OR “RTW”

“Subjective well-being” OR “Life Satisfaction” OR “Return to work” OR “RTW”

“Job satisfaction” OR “Well-Being” AND “Return to work” OR “RTW” AND “Work–health incompatibility”

“Subjective well-being” OR “Life Satisfaction” AND “return to work” OR “RTW” AND “Work–health incompatibility”

“Job satisfaction” OR “Well-Being” AND “Return to work” OR “RTW” AND “Job retention factors

“Subjective well-being” OR “Life Satisfaction” AND “Return to work” OR “RTW” AND “Job retention factors

### 2.5. Study Selection

Firstly, duplicated records were removed. During an initial phase, all articles were assessed based on title and abstract. If the title or the abstract failed to comply with one or more of the selection criteria, the publication was eliminated from the review. In the event of the title or abstract providing insufficient information for determining the article’s eligibility, the full text was assessed.

Finally, all articles which complied with the selection criteria were selected. These criteria were as follows:

(1) It must be an academic article.
(2) It must be written in Spanish or English.
(3) Participant characteristics: the sample must be comprised of adult employees.
(4) Admissible study designs: qualitative, correlational, and quasi-experimental.
(5) Construct being researched: the article must assess employees’ subjective well-being at work or job satisfaction after their return to work following a serious health problem.
(6) Time range: no studies from before the year 2000.

Exclusion criteria were (in addition to a failure to comply with the inclusion criteria outlined above): articles published in a conference communication format, books, theses, reviews, dissertations, or articles published in popular science magazines.

### 2.6. Data Collection Process

To gather the necessary data, a form was designed to enable the data from the studies included in the systematic review to be extracted, summarized, presented, and critically evaluated.

### 2.7. Data List

Once the articles that complied with the established eligibility criteria had been selected, a coding manual was designed, along with a protocol for registering the characteristics of each study. The aim was to describe the moderator variables under a set of specific criteria, to guarantee the transparency and replicability of the coding process. Thus, an ad hoc scale was compiled following Rubio-Aparicio et al.’s recommendations [61]. The scale contained three general categories:

- Methodological variables: these refer to the type of design used, and the research methods applied during the studies, the quality of the measures carried out and the procedures followed for the data analysis. This category encompasses the following variables:
  A.1. Size of the sample.
  A.2. Sampling process (1: random; 2: convenience; 3: volunteers).
  A.3. Assessment instruments used to evaluate subjective well-being and/or job satisfaction (measures used).
  A.4. Dimensions or variables included in the assessment instrument (1: work–health balance; 2: subjective well-being; 3: job satisfaction).
  A.5. The design used (1: qualitative; 2: quasi-experimental; 3: correlational).
  A.6. Data collection (1: online or by conventional mail or telephone; 2: at the workplace or in the interviewees’ homes).
- Substantive variables: these refer to the sociodemographic characteristics of the sample and the characteristics of the treatment, as well as to the context in which the research took place. This category encompasses the following criteria:
  B.1. Mean age of the sample.
  B.2. Percentage of women.
  B.3. Percentage of the sample living with a life partner.
- Extrinsic variables: these refer to those characteristics which have nothing to do with the object of study, but which may be associated with the results. Those included were:
  C.1. The status of the publication (1: published; 2: unpublished).
  C.2. Year in which the article was published.
  C.3. Type of publication (1: journal included in the Journal Citations Reports (JCR); 2: journal not included in the Journal Citations Reports (JCR).
  C.4. Information about the lead author: gender (W: woman; M: man) and affiliation (institution to which they belong).
  C.5. Country in which the study was carried out.
  C.6. Funding source.

### 2.8. Risk of Bias in Individual Studies

No bias risk assessment was carried out for the studies included in the review, since the issue has been studied very little to date and the number of available studies was limited.

### 2.9. Summary of the Results

Probably as a result of there being some lack of clarity remaining regarding how well-being at work should be defined and measured [62], the studies that focus on this construct are extremely varied, with substantial differences between them. It is, therefore, neither possible nor appropriate to make valid comparisons between the different studies since the definitions of the variables and the instruments used vary considerably. Given these circumstances, it was not possible to carry out a quantitative synthesis of the data, and we will therefore focus instead on presenting the results of each study individually (i.e., the synthesis carried out was qualitative).

## 3. Results

### 3.1. Article Selection

**Figure 2:**
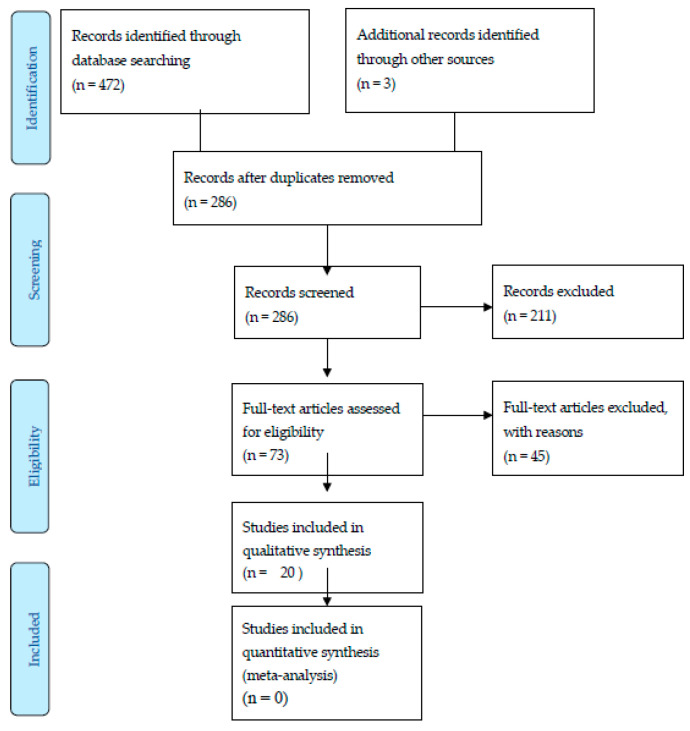
Flow diagram of the article selection process.

The process outlined above returned 472 articles of potential interest located in electronic databases, plus another 3 articles identified by other sources (consultation with expert academics), as Figure 2 shows. This number was reduced to 286 after duplicates had been removed. After reading the title and abstract, 73 studies were chosen for full-text analysis. Subsequently, we excluded those articles that:

(1) Were not electronically enabled.
(2) Presented only the theoretical framework.
(3) Presented only design, with a said design never actually being implemented.
(4) Focused on improvements in quality of life and the overall well-being of people returning to work but did not specifically assess their well-being at work or job satisfaction after their RTW.

The final sample for the systematic review comprised 20 studies.

Figure 1 presents a flow diagram that summarizes the study screening and selection process, including those articles that were finally included in the review, as well as those that were excluded.

### 3.2. Sociodemographic Data

The information regarding points B1, B2, and B3 were summarized from the total sample of 7387 subjects. The mean age (48.31), mean percentage of females in the studies (37.16%) and mean percentage of the sample living with a life partner (69.85%) were calculated globally, even though this information is missing in some studies. All the subjects in the sample complied with one of the following requisites: they had an LSHPD or a chronic illness, or they had recovered from a serious or moderate illness or injury.

### 3.3. Study Characteristics

With regard to the extrinsic variables, the following aspects should be highlighted: all 20 studies selected for the review were published in English between 2004 and 2019. All were published in journals included in the Journal Citations Reports. Funding sources were specified, with some exceptions, such as the study carried out by Abegglen et al. [62], which was supported by the Swiss Accident Insurance Fund. The origin of the sample was coded based on the information provided in the description of the samples, with the majority of the research conducted in the United States of America and Europe, while several studies were from Brazil or Japan. The universities to which the lead researchers belonged can be consulted in Table 1. Nine of the lead researchers were men.

With regard to the methodological variables, in all cases a convenience and/or volunteer sampling procedure was used. To assess employees’ subjective and psychological well-being (job satisfaction) at work, the studies analyzed used a wide variety of different designs, methods, and evaluation instruments. Each study used one or more evaluation instruments to assess different aspects related to well-being at work following RTW. The diversity of instruments, as well as the dimensions or variables, used to determine employee well-being after RTW highlights the lack of clarity regarding how this concept should be conceptualized and measured. With regard to design, two studies were qualitative [63,64], the study by Yonezawa et al. [65] can be considered quasi-experimental and the remaining seventeen were correlational. The data were gathered online, by conventional mail or over the telephone (five studies), in person at the workplace (seven studies), or in interviewees’ homes (two studies). The studies by Miglioretti et al. [66] and Royal et al. [63] collected data online, by conventional mail, over the telephone, and in respondents’ homes. Four articles failed to specify how exactly the data collection process was carried out.

In the following section, we will outline in more detail the basic data extracted from each of the 20 studies included in the review. All of the selected articles were focused on well-being at work and job satisfaction after a serious health problem [67,68,69,70].

### 3.4. Well-Being at Work, Job Satisfaction and Other Work-Related Outcomes after a Serious Health Problem

First, with respect to some instruments, the Work and Health Questionnaire (WHQ) has been presented [71] as a valid and reliable tool for identifying workers with moderate injuries at risk of complicated rehabilitation. The WHQ encompasses five factors: workplace, RTW cognitions, pain, Post Traumatic Stress Disorder symptoms, worries, and anxiety. A “complicated rehabilitation” is defined as one resulting in more days of inability to work, a decrease in life satisfaction, an increase in negative feelings (subjective well-being), and diminished job satisfaction (psychological well-being). Analyses of the predictive capacity of the WHQ revealed significant associations between its subscales and length of inability to work, job satisfaction, and RTW. These results reaffirm the importance of psychopathological, cognitive, social, and occupation assessment and confirm the biopsychosocial nature of inability to work and RTW. Job support and job strain were significantly associated with job satisfaction 18 months after injury, while job design predicted days of inability to work. Only the Anxiety/Worries subscale of the WHQ was found to significantly predict employee subjective well-being in terms of negative feelings and life satisfaction. In a similar line of research, Gragnano et al. [4] presented the Work–Health Balance Questionnaire (WHBq), a valid instrument for studying and predicting the work retention of employees returning to work with LSHPD. Work–Health Balance refers to workers’ management of their work demands and health needs. The instrument measures three factors: work–health incompatibility, healthy climate, and external support. These dimensions are in turn associated with psychological well-being in the workplace, dysfunctional behaviors at work, and general psychological health. A higher level on the work–health balance index is linked to lower levels of presenteeism, emotional exhaustion, workaholism, and psychological distress, as well as to higher levels of job satisfaction and work engagement.

Considering job satisfaction among those who return to work, Bush et al. [71] found that following a traumatic brain injury, the job satisfaction may be associated more with participation in productive activities than with monetary compensation (psychological well-being). Furthermore, employment factors seem to be related to social aspects affecting positively a person’s quality of life and general (subjective) well-being. Moreover, they concluded that, due to the idiosyncratic nature of each injury and recovery process, it is necessary to study each case individually to adapt to each survivor’s unique life circumstances. In another study, carried out in 2015, Fiabane et al. [72] found that the best predictors of a satisfactory RTW following an invasive cardiac procedure were: baseline job satisfaction, depression, and ambition, which were independent of sociodemographic and medical factors. Moreover, cardiac patients who had a partial RTW reported higher levels of job satisfaction than those who had a full RTW, regardless of their baseline job satisfaction (psychological well-being). Finally, other findings [24] supported that job satisfaction increased with the number of worked hours of those who RTW. Near a third of participants who were unemployed after the stroke were satisfied with their situation, for instance, a voluntary job or a formal education.

Current work ability emerges as a reliable predictor for RTW in recent studies. Following this approach, de Boer et al. [29] found that it improves significantly from 6 to 18 months after the first day of sick leave among cancer survivors. While the work ability seems to be severely damaged in the first months, it improves significantly afterward.

Work stress and some correlates, such as high job demands and low resources at work, emerge as relevant factors among the findings of the empirical studies. For instance, Endo et al. [73] concluded that, among patients who have reported sick due to depression, high organizational job demand (evaluated using the Brief Job Stress Questionnaire, BJSQ) is a risk factor for recurrent sickness absence after RTW. The Brief Job Stress Questionnaire is a multidimensional job stress questionnaire comprising 57 items. It contains questions linked to job stress factors (17 items), stress response (29 items), social factors (9 items), and job and life satisfaction (2 items). The Brief Job Stress Questionnaire can be used as a mental health check, as well as to assess and improve the psychological work environment (psychological well-being). In the same vein, another empirical study by Fiabane et al. [74] explored changes in self-reported psychological health and perceived work stress among patients returning to work following heart surgery. The main finding reported by the study was that cardiac patients experienced an improvement in subjective psychological health and did not perceive increased work stress after their RTW.

Nevertheless, they also observed a decrease in job satisfaction levels (psychological well-being). Finally, with an expanded model, the aim of the study carried out by Yonezawa et al. [65] was to determine the effect of phase II cardiac rehabilitation on job stress and health-related quality of life after return to work in a sample of middle-aged patients with acute myocardial infarction. Job stress was assessed using a brief job stress questionnaire containing questions related to job stressors, worksite support, satisfaction with work or daily life (psychological well-being), and psychological distress. Health-related quality of life was evaluated using the short-form 36-item Health Survey (SF-36). Patients in the cardiac rehabilitation group scored significantly higher for job stressors and psychological distress and had higher SF-36 scores six months after acute myocardial infarction than their counterparts in the non- cardiac rehabilitation group.

Related to satisfaction with life, Vestling et al. [75] found that participants who had returned to work after experiencing a stroke reported significantly higher levels of satisfaction concerning their professional situation and significantly greater subjective well-being about work than their counterparts who never returned to work.

### 3.5. Potential Moderator Role of Gender and Organizational Policies and Its Impact on Career Development

Some approaches propose that gender (at the personal level) or firms’ policies (at the organizational level) could exert a moderator role in the relationships between RTW and outcomes. Even though more research is needed to clarify this role, a pair of studies relied on these ideas. Related to gender differences, on the one hand, one of the main results reported by De Rijk et al. [70] in their study among those with long-term illnesses connected with some work modifications (working reduced hours and reducing the pace of work) during the return-to-work process. On average, women experienced more job satisfaction (psychological well-being) than men following their return to work after reporting sick for more than one month due to mental or musculoskeletal disorders (psychological well-being). On the other hand, Arndt et al. [27] failed to find any differences in the probability of RTW among cancer survivors concerning their gender. In addition, with respect to organizational practices, Huang et al. [76] explored how organizational support and return-to-work policies are associated with post-injury job satisfaction (psychological well-being). Organizational support was found to be an important predictor of post-injury job satisfaction, as was the quality of return-to-work policies.

With respect to the career development of those who RTW, one study carried out with a spinal cord injury cohort [77] sought to determine whether patients had returned to their pre-injury employer, had changed employers, or had never returned to paid work post-spinal cord injury. The authors measured subjective well-being using three indicators: income, quality of life, and life satisfaction. The results revealed no significant differences in terms of subjective well-being among those who had returned to their pre-injury employer and those who had changed employers. Additionally, job satisfaction (psychological well-being) at the time of a spinal cord injury onset is associated with a shorter RTW time, whereas a traumatic spinal cord injury etiology prolonged it. In the same vein, a multi-regional study from Germany showed that 63% of all cancer survivors returned to their old job, while 7% changed it. These findings seem to be confirmed by Marom et al. [26], who found that 90% of the returned workers had the same position in the same workplace. Despite these findings, some authors suggested that the majority of the research on the impact of the disease on employment status has been conducted immediately after the diagnosis, while the long-lasting impact of illness has been neglected. Almost all of the women survivors of breast cancer lost their jobs or suffered a significant reduction in working hours [25].

### 3.6. Different Methodological Approaches

Some studies adopt the strategy of comparison among the situation before and after the disease. First, Killey et al. [78] compared pre- and post-job satisfaction rates in a sample of stroke victims. Following their return to work, participants were significantly less satisfied with their job suitability (physical, cognitive, and financial), stability, and importance. Participants reported impacts at work due to residual disability (which affected their work performance, including fatigue, concentration, mobility/walking, physical strength, and memory), changes in working habits, and reduced job satisfaction (psychological well-being). In a related way, Miglioretti et al. [66] focused on the quality of work experience before an angioplasty or heart surgery and after RTW. The main finding was that participants experienced a generalized deterioration in the quality of their work experience, linked to different aspects of the work experience that were perceived by participants as having worsened. In this study, the variables used to measure the quality of work experience were job involvement and job satisfaction (psychological well-being). Both were found to decrease after RTW in a statistically significant manner, dropping by 64% (job involvement) and 59% (job satisfaction). The unique study with a Latin American sample [30] showed that 44% of the participants returned to paid work during the first six months, while among those with severe consequences from the disease, only 32% returned to work later. Detrimental consequences on financial security and the number of paid hours were also reported.

From a different methodological approach, one the qualitative study [64] explored the experiences of people who returned to work after being diagnosed with and having recovered from Guillain-Barré syndrome, in order to gain insight into the factors which facilitated or inhibited this process. The interviews revealed that after returning to work, participants continued to experience certain physical and psychosocial difficulties related to the Guillain-Barré syndrome, which required active coping strategies. Working was found to have a positive effect on the general subjective well-being of those returning to work, in as much as they place a high value upon the process of returning to work as a very significant step towards moving forwards with their lives, and recovering health, social relationships and a normal self-image. Nevertheless, it also increased their concerns regarding the stigma attached to the disease and their worries about being different. Consequently, participants’ opinions about adaptations at work and the support received from their colleagues and managers (psychological well-being) were ambivalent, since although they recognized and acknowledged their practical usefulness, they were fearful of the associated stigma. Moreover, Pomme et al. [25], also with a qualitative study, found that the participants were uncomfortable talking about their diagnosis and treatment with people at work, and at the same time, those who were unemployed reported that they do not inform about their diseases to the potential employers.

## 4. Discussion

### 4.1. Summary of the Evidence

In recent years, employee well-being has played an increasingly prominent role in health research. Although research into well-being at work is a broad domain which has flourished in recent decades [49], RTW research does not specify clearly its definition or measurement [50]. Alongside this, there is pressing social concern and a growing interest among academics regarding general RTW after health problems [79].

The studies reviewed in the present article focus on subjective and psychological well-being at work following a health problem. The aspects taken into account to assess and measure employees’ subjective experiences serve to highlight the lack of clarity regarding what exactly well-being in the RTW process is and how it should be measured.

The systematic search conducted here returned 20 articles. The qualitative analysis of the studies included in the review highlights the lack of clarity regarding the concept of well-being at work and how it should be measured. Studies focusing on well-being at work after RTW referred to stroke [78,80], traumatic brain injury [63], mental or musculoskeletal disorders [72], depression [73], heart problems [65,66,74], musculoskeletal injuries [76], spinal cord injury [77] and Guillain-Barré syndrome [64], among others.

Our results partially match with those from De Jong et al. [79]. They carried out a systematic review to assess factors that contribute to the quality of working life of employees with a chronic physical disease. They identified five factor categories, including individual work perceptions. This category, in turn, encompassed the following themes: job satisfaction, job commitment, work values, job motivation, feelings about the current job, and emotional challenges. The search carried out by De Jong et al. did not specifically include the term well-being at work, which is why our review may to some extent complement theirs. In a similar vein, Greidanus et al. [81] systematically reviewed 47 studies highlighting employer vs. employee perceptions of work participation of cancer survivors. Since our review confirms their results regarding the facilitating role of social support on employees’ willingness to RTW, their findings were more focused on the discrepancies between the workers and the employers’ points of view.

Gragnano et al. [4] conducted a review of reviews, in an attempt to summarize all the common factors that preclude RTW among different diseases. Hence, our findings also support their general conclusion that a wide range of psychosocial factors seems to be potential common predictors of RTW for different kinds of diseases. Related to this point, our findings also support that perceived stress (including high demands and few resources) could explain low rates of RTW, mainly among specific groups characterized by old age, low education, and low socioeconomic status. As research widely suggested [82], the relationships between work and health seem to be circular. This means that those with good health could obtain and maintain better jobs, while better jobs also affect health, by providing higher income, better health insurance, and living conditions.

#### 4.1.1. Theoretical Approaches

Our results can be summarized under the three different theoretical approaches for understanding RTW and its antecedents and consequences. The first is focused on the relationships between the two dimensions of well-being. The second is devoted to the identification of the indicators of health or illness. In addition, the third is aimed at clarifying the relationships between RTW and quality of life. With respect to the association between psychological and subjective well-being, data suggest that RTW has a positive effect on job satisfaction [63,80], mental, social relationships, and self-image and subjective well-being [64]. More specifically, autonomy at work increases life satisfaction and decreases negative feelings [67]. Although the empirical evidence seems to be scarce [26], the mediating role of personal characteristics, as self-efficacy, in the relationships between predictors and RTW deserves more attention.

Regarding the physiological indicators of health or illness, our results show that higher psychological job demand is linked to more risk for sickness absence [73], less job satisfaction [65,74] (mainly, with a full RTW [83]), and a deterioration in the quality of the work experience [66]. Nevertheless, work modifications during the return-to-work process reduce physiological symptoms and increase job satisfaction, especially in the case of women [72]. Furthermore, a higher level of work–health balance is associated with higher levels of presenteeism and workaholism, as well as job satisfaction and work engagement [75]. It is also important to stress that a residual disability or a complicated rehabilitation negatively affects work performance, physical health (e.g., fatigue, concentration) as well as diminishes job and life satisfaction increases negative feelings [71,78].

Concerning the relationships between RTW and the quality of working life (e.g., interpersonal relations, levels of social support), our results suggested that supervisor support is linked to job satisfaction [62,76,77] in the case of a disease. Despite this empirical evidence, the psychosocial meaning of support should be considered with caution. As research has suggested [84], social support could be considered beneficial if it meets personal needs, but could also be viewed as damaging. As previous research suggests caution regarding the potential threat that social support can exert on personal well-being [85], we would recommend not to suggest interventions for increasing social support as a universal resource.

#### 4.1.2. Questionnaires

With regard to measurement instruments, a few studies analyze the relationship between psychological (job satisfaction) and subjective (life satisfaction, positive and negative affect) well-being and different organizational variables (e.g., the Work Adjustment Scale and the Environment scale). These studies are more frequently linked to the first approach, which evaluates the different dimensions of well-being.

The second theoretical approach uses manifold scales to measure stress and physiological indicators of health [66,68,72], although some of them are purpose-designed questionnaires [74,78]. The most used are the Center for Epidemiologic Studies Depression Scale (CESD) [28,69] and the Brief Job Stress Questionnaire [65,73]. It should also be mentioned that Abegglen et al. [62] and Gragnano et al. [83] presented two recently developed scales: The Work and Health Questionnaire and the Work–Health Balance Questionnaire (WHBq). The former identifies workers at risk of a complicated recovery process. The scale has five underlying factors (Job Design, Work Support, Job Strain, Somatic Condition/Pain, and Anxiety/Worries) measuring different aspects of work and health-related risk factors of injured workers. Although the WHQ offers good psychometric properties and appears to be suitable for exploring relations with psychological (job satisfaction) and subjective well-being (life satisfaction and negative feelings), it has only been tested with workers with minor to moderate accidental injuries. On the other hand, the WHB index focuses on the adjustment between health needs and work demands. The scale includes three factors (work–health incompatibility, healthy climate, and external support) and shows good psychometric characteristics and strong consistent relationships with psychological well-being (e.g., job satisfaction, work engagement), dysfunctional behaviors at work (e.g., presenteeism), and general psychological health. Unlike the WHQ, a few studies have confirmed the utility of a single index of WHBq with aging employees, as well as, its predictive validity [82,86].

Regarding the third theoretical approach, these studies use scales elaborated by authors [62,76,77] to measure the quality of life, the work–life balance, or (supervisor) support. All of them show a significant correlation with subjective and psychological well-being.

Finally, empirical research on RTW continues its advancement, as the plethora of studies published during 2020 shows [87,88,89,90], while the systematic reviews and the methodological quality analysis of the existing literature [91] also contribute to expanding our comprehension of the RTW phenomenon as the main threat for the integration of people with health conditions in developed countries [5].

In sum, each of the studies included in the review adopts a different theoretical framework and, as a result, uses different instruments to assess well-being at work after RTW. This renders any comparison between the different studies both unfeasible and inadequate. Nevertheless, the following conclusions can be drawn from the qualitative analysis carried out:

- The results indicate a higher level of (psychological and/or subjective) well-being among those who return to work following a health problem.
- Those who return to work may find themselves reassessing their priorities, attaching greater importance to looking after their health.
- Employees with a physical health problem (with residual symptoms) may experience reduced (psychological and/or subjective) well-being at work due to health-work.

**Figure 3:**
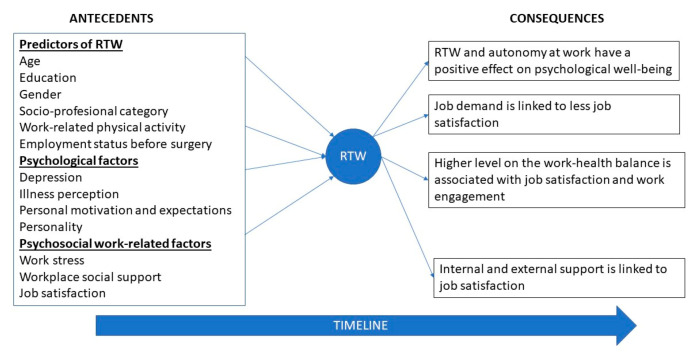
Main findings of the review.

**Figure 4:**
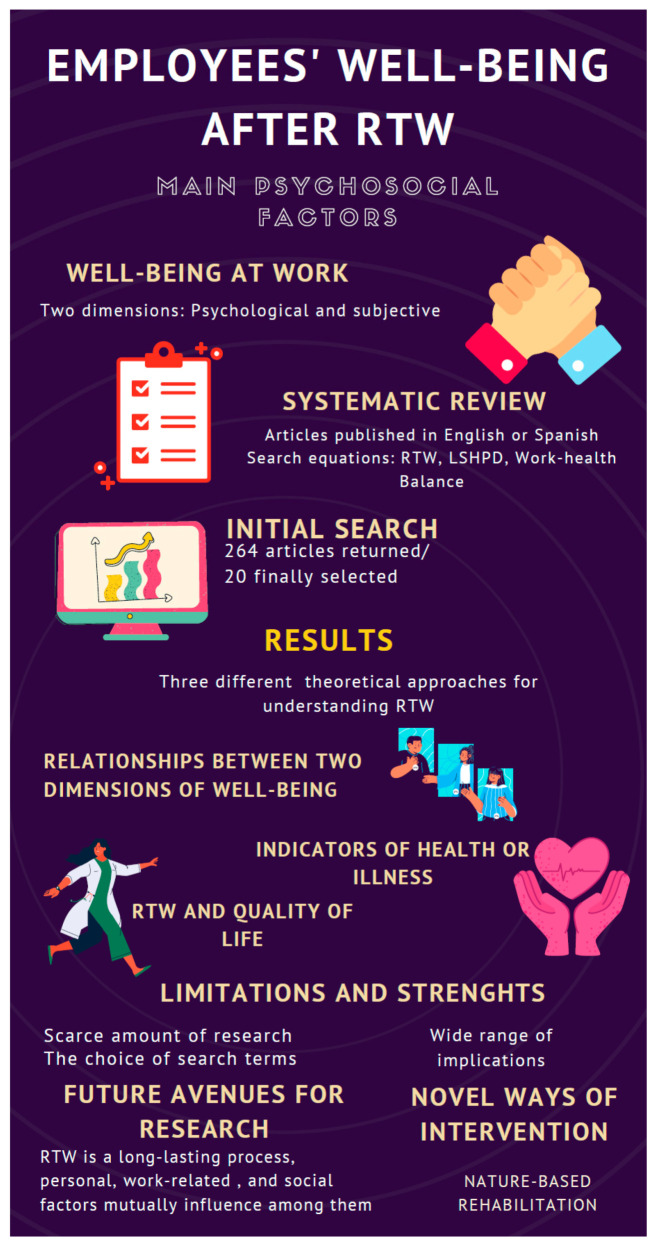
Graphical abstract.

Some of the relationships supported by our findings have been summarized in Figure 3 and Figure 4.

### 4.2. Limitations, Suggestions for Future Research, and Practical Implications

This systematic review of the literature is exploratory and includes both qualitative and quantitative studies. This cross-disease approach enables a broad description of well-being at work after RTW.

The main limitation of this systematic review is the scarce amount of research that has been carried out into well-being at work after RTW. The studies included in the review approach the concept from different perspectives and, as a result, measure well-being using different methods and tools. Only a very few studies assess subjective and psychological well-being at work and the majority of these evaluate factors that contribute to said well-being or are associated with it in one way or another, such as autonomy at work, job stress, and depressive symptoms, among others. This means that they cannot, and indeed should not be compared. Thus, one of the aspects that must be harmonized to improve future research into well-being at work after RTW is how to measure this particular type of well-being.

Another limitation may be the choice of search terms. Our search may have been too restrictive, causing us to overlook certain studies which may have contributed to the general description of employees’ well-being at work after RTW. Moreover, only published scientific articles were included in the review, and given the novelty of this issue and the increasing interest it is generating, we may have overlooked some important contributions.

The process by which we synthesized the results may also be a limitation. Although we only selected articles that explicitly mentioned well-being at work or job satisfaction after RTW, not all of them aimed to explore well-being at work per se, and some associated it with other aspects of work. Nevertheless, the subjective well-being at work of employees who return to work after recovering from a health problem is an issue that should be deeply explored due to its implications for individuals, families, and societies. Thus, we believe that this systematic review makes a meaningful contribution to the literature since it suggests aspects that may be important for this construct.

The present study also has some strengths, such as the inclusion of a wide sample of empirical studies assessing employees’ subjective well-being at work or job satisfaction after their return to work following a serious health problem. Even though there are a lot of publications that include RTW as a keyword or as a central idea, there are fewer papers that include an empirical assessment of both constructs among the specific type of workers of interest.

Considering the future avenues for research, some authors suggested that facilitators and impediments to RTW could change over time [25], as long as the labor market evolves and job features are affected by globalization and technology. On the positive side, for instance, the current Covid-19 pandemic showed that the way by which the activities are developed could rapidly change, allowing more teleworking and reducing the time devoted to traveling to our workplace. On the negative side, technology is omnipresent at work, and illness survivors need to constantly adapt by learning new abilities. In this sense, their cognitive impairments could be considered threatening, precluding them from fast adaptation to their jobs. On a personal level, some individual features could be affected by severe illness, also affecting RTW, such as occupational future time perspective and death anxiety. When confronting death and illness, people could change their perception of time, including the length of future time and their opportunities and barriers, and this occupational future time perspective has been shown to be a predictor of intention to remain at work and desires to retire early [92]. Additionally, death anxiety has been defined as a complex emotional response triggered by illness and the proximity of death. As previous research has shown [93], death anxiety could impinge on a lot of life spheres, like consumer behavior, finances, and also work-related behavior. In this vein, future studies could deeply explore the potential role of these variables in the willingness of RTW among survivors. Moreover, the relationships between RTW and sickness absence would be complex, and are thus deserving of future research. The effort invested in working could be associated with higher sick leave, as previous studies have shown [94]. For instance, those workers that RTW after an absence associated with stress could experience higher occupational stress due to organizational pressures or decreased coworkers’ support. Finally, gender differences should also be considered in a more detailed manner. First, women are very frequently affected by specific diseases like breast cancer with a strong impact on their health, but also their careers. Second, the impact of illness on women’s career development could occur while they are young, due to pregnancy and child-care obligations, but also during mature age due to the eldercare–work conflict.

Our research also has strong implications for intervention. First of all, our findings highlight that the RTW is a long-lasting process, where personal, work-related, and social factors mutually influence among them. Hence, the interventions aimed to promote RTW should be multidisciplinarily oriented [95] and open to novel ways of intervention, like nature-based rehabilitation [36]. Secondly, our findings should be considered under the diversity management point of view. As organizations should retain older and more highly qualified workers [96], and aged workers could also be more frequently affected by diseases, firms should adapt themselves to offer a friendly environment to those that return to work from illness. In a related vein, the present evidence underlines the relevance of interventions that increase both organizational and family support for those workers that return to their jobs after an illness.

## 5. Conclusions

Given the current configuration of the labor market, as well as the importance of well-being at work for facilitating the successful and sustained RTW of older employees, those with health problems, this systematic review may constitute a good starting point for managing these labor force groups (see Table 1 for more details). Nevertheless, the accumulated knowledge about well-being at work after RTW is fairly poor, and it is difficult to draw reliable conclusions based on the scientific evidence presented by the research carried out to date.

It is important to provide human resource managers, researchers, and health professionals with the tools they need to manage and monitor the RTW and WR processes of employees with chronic health problems. It would therefore be interesting to carry out more studies on employees’ subjective and psychological well-being after RTW, as well as on the factors that contribute to this optimum state since this would help foster successful and sustained RTW, which has benefits not only for the individual him or herself but also for employers and society in general.

## Figures and Tables

**Table 1:** Studies included in the review.

	Methodological Variables					Substantive Variables			Extrinsic Variables		Main Findings
Article	A.1	A.3	A.4	A.5	A.6	B.1	B.2	B.3	C.4	C.5	
1. Abegglen et al. 2017 [62]	1963	Work and Health Questionnaire (WHQ) Bern Questionnaire on Well-Being (BQW) A single item of the Short Job Satisfaction Questionnaire (AZK)	Workplace characteristics, RTW cognitions, pain, PTSD symptoms, worries, and anxiety. Subjective Well-being (life satisfactions and negative feelings) Job-related well-being (psychological well-being)	3	2	46.78	23.6	n.a.	W/University of Bern	Switzerland	Adequate Psychometric properties of the WHQ, and high clinical utility to predict days of work disability among injured workers.
2. Arndt et al. 2019 [27]	1558	Detailed questions regarding changes in employment status since the time of diagnosis	Different indicators of well-being such as whether and when there had been changes such as uptake of a new job, reduction of working hours, vocational retraining, unemployment, disability pension, early or old-age retirement	3	1	50.1	75	74.6	M/German Cancer Aid	Germany	Long-term survivors after cancer are able to RTW. Financial problems might arise due to a reduction in working hours. Probability of RTW was strongly related with age at diagnosis, tumor stage, education, and occupational class but did not differ with respect to the tumor site, gender nor marital status.
3. Bush et al. 2016 [71]	5	Interviews, semi-structured	Three major conjectures regarding the return to work experiences of adults with Traumatic brain injury (TBI): (a) Job Satisfaction (Psychological well-being) (b) Cognitively demanding careers (c) Job modifications and strategies	1	2	49.2	40	n.a.	W/University of Wyoming	USA	Job satisfaction may relate more to involvement in productive activities than monetary compensation; Adults with TBI can be successful in holding and maintaining positions with high cognitive demands Individualized job modifications and strategies are likely necessary for adults with TBI to succeed vocationally.
4. De Boer et al. 2008 [28]	195	Work Ability Index (WAI) Dutch Questionnaire on Experience and Judgement of Work (VBBA).	Current work ability compared with their lifetime best. Current physical and mental work ability in relation to job demands. Physical workload and work stress	1	2	42.2	60	82	M/Dutch Cancer Society	The Netherlands	Work ability at 6 months strongly predicted RTW at 18 months, after correction for the influence of age and treatment. Self-assessed work ability is an important factor in the return-to-work process of cancer patients independent of age and clinical factors.
5. De Rijk et al.2009 [70]	119	Maastricht Autonomy Questionnaire (MAQ) Questionnaire work and health/Questionnaire (VBBA) (in a Dutch translation) Job Content Questionnaire (in a Dutch translation) Job satisfaction (one item)	Job autonomy: (a) Emotional demands, (b) opportunities for learning and (c) career development, commitment. (a) Psychological work demands, (b) Support from colleagues, (c) Support from supervisor. Job satisfaction (Psychological well-being)	3	1	D	45.37	68.96	W/Maastricht University	The Netherlands	Work modifications were widely applied during the RTW process and predominantly aimed at reduction of pressure at work. Women had a few more work modifications. The marginal gender differences may be due to male and female respondents having similar characteristics. Upon RTW some job characteristics improved.
6. Endo et al. 2015 [73]	540	Brief Job Stress Questionnaire (BJSQ) (psychological characteristics of the work environment)	Job stressors, stress response, social factors and work and life satisfaction (Psychological and Subjective well-being): (a) Job demand and (b) job control	3	2	41.7	15.74	77.59	M/Dokkyo Medical University School of Medicine	Japan	High organizational job demands is a risk factor for recurrent sickness absence due to depression after RTW.
7. Fiabane et al. 2015 (a) [72]	108	State Trait Anxiety Inventory Depression Questionnaire Constructed Meaning Scale Occupational Stress Indicator (OSI)	Anxiety Depression Illness perception Source of stress, (b) Type A Behavior (c) Locus of control (d) Coping strategies (e) Job satisfaction (f) Mental Health (g) Physical Health (Psychological well-being)	3	2	49.2	16.4	70.5	W/University of Pavia	Italy	Cardiac patients had an improvement in subjective psychological health and did not perceive increased work stress after their RTW. Patients’ psychological health and work stress need to be assessed during the cardiac rehabilitation and should be also carefully monitored after the RTW.
8. Fiabane et al. 2015 (b) [74]	90	Occupational Stress Indicator (OSI) State Trait Anxiety Inventory (STAI-X1) Depression Questionnaire (DQ) Constructed Meaning Scale (CMS)	Source of stress, (b) Type A Behavior (c) Locus of control (d) Coping strategies (e) Job satisfaction (f) Physical Health (Psychological Well-being) Anxiety Depression Illness perception	3	1	49.33	8.3	72.2	W/University of Pavia	Italy	Baseline job satisfaction, depression and ambition turned out to be independent, significant predictors of job satisfaction following RTW.
9. Gragnano et al. 2017 [83]	321	Work–Health Balance Questionnaire (WHBq) Psychological well-being at work. a) Job satisfaction single item. b) Italian version of the Utrecht Work Engagement Scale (UWES-9). c) The emotional exhaustion scale of the Maslach Burnout Inventory General Survey (MBI-GS; Italian version: Dysfunctional behavior at work: a) Single item by Aronsson, Gustafsson, and Dallner, 2000). b) DUWAS; Italian versión) General psychological health. Italian version of the General Health Questionnaire (GHQ-12)	Adjustment Between Work Demands and Health Need Job satisfaction Work engagement Emotional well-being at work. Feelings of being emotionally strained and tired by one’s work Presenteeism *Workaholism:* working Compulsively and working excessively Psychological distress experienced within the last 2 weeks	3	1	45	55	n.a.	M/ University of Milano Bicocca	Italy	A higher level on the WHB index was associated with lower levels of presenteeism, emotional exhaustion, workaholism, and psychological distress and with higher levels of job satisfaction and work engagement, supporting the construct validity of the instrument.
10. Huang et al. 2004 [76]	1438	Scale elaborated by the authors: a) Organizational support b) Quality of return to work polices c) Job satisfaction	Organizational support (5 items) Quality of return-to-work policies (6 items) Post-injury job satisfaction (6 items) (Psychological Well-Being)	3	2	n.a.	27.7 Construction workers n.a. for Trasportation workers	n.a.	W/*Liberty Mutual Research Institute for Safety*	USA	Factor analyses supported the two-factor structure of the scale, and both organizational support and RTW policies were independently associated with post-injury job satisfaction.
11. Killey et al. 2014 [78]	21	Purpose-designed questionnaire to measure Stroke-related Characteristics of Participants a) Hospital length of stay b) Time between hospital discharge and work return Common problems after stroke impacting work	The questionnaire was developed to elicit stroke-related demographics and to gather information from participants that has been highlighted as important from previous literature regarding return to work (Concentration, problem solving, interacting with others). The job satisfaction questions were developed for the purposes of this study (Psychological Well-being)	3	1	48	67	n.a.	W/*University of Queensland*	Australia	Participants did not routinely access formal support services to RTW, while experiencing changes to work-related habits and satisfaction.
12. Marom et al. 2018 [26]	178	Perception of self-efficacy Job Content Questionnaire Impact of Event Scale World Health Organization Disability Assessment Schedule 2.0 Disability of Arm, Shoulder and Hand (DASH) Outcome Measure Self-reported assessment of function and disability Self-reported assessment of extent of mental disorder	Capability of the person to participate in work, social activity, and activities that require mobility and general functioning Self-reported assessment of occupational stress based on psychosocial factors, job demands, control, support model 3-part questionnaire: workload, job control, social support Mental disorders: intrusive thoughts and patterns of avoidance related to the hand injury. Function and disability:capabilities in 6 domains: cognition, mobility, self-care, interpersonal interactions, life activities, and participation in society, mapped onto the International Classification of Functioning, Disability and Health	3	1	37.4	0	n.a.	M/ Occupational Therapy Units of the Clalit Health Services	Israel	RTW was determined by the physical capability of the hand, pain, and psychosocial factors, but it was also affected by legal factors. Participants who did not RTW during the first 9 months are at risk for long-term disability.
13. Miglioretti et al. 2018 [65]	137	Job Involvement Questionnaire Job Content Questionnaire Four items that analyzed global Satisfaction Hospital Anxiety and Depression Scale Brief Illness Perception Questionnaire Adherence Schedule in Heart Disease	Quality of working life Psychosocial characteristics of perceived work conditions (psychological job demands, physical job demands, decision latitude; supervisor and co-worker support) (psychological well-being) Job satisfaction (psychological well-being) Psychological health: anxiety and depression The patients’ perception of disease Severity Self-efficacy in the management of stressful situations and self-efficacy in adherence to medical prescriptions	3	1&2	51.9	14	70	M/ Università degli Studi di Milano-Bicocca	Italy	Work hours, job satisfaction and job involvement significantly decreased after RTW in angioplasty or heart surgery survivors.
14. Nascimento et al. 2019 [29]	97	Modified Rankin Scale (mRS) Hospital Anxiety and Depression Scale	Independence in activities of daily living Depression	3	2	57	44	64	M/Conselho Nacional de Desenvolvimento Cientıfico e Tecnologico	Brasil	Contribution to household income, being a white-collar worker and being independent in daily activities at 3 months, positively predicted RTW. Less than 50% of stroke survivors returned to work six months after stroke. Among predictors, only the level of dependence in daily activities is a modifiable factor
15. van Maarschalkerweerd et al. 2019 [25]	19	Focus group interviews	List of topics describing the participants’ perceptions about how her work had changed after being diagnosed with breast cancer.	1	2 (at the hospital)	49	100	52	W/ Pink Ribbon Foundation and Dutch Cancer Society	The Netherlands	Breast cancer survivors still experience changes in employment status 5–10 years after diagnosis. Perceived barriers to RTW shortly after breast cancer diagnosis tended to be disease- and treatment-related, while 5–10 years later, they were personal- and work-related.
16. Royal et al. 2009 [63]	5	Experiences of adults returning to work by an in-depth interview	Interpretative phenomenological analysis. Issues (psychological well-being): a) Perceived value of work b) Work both challenges and restores a familiar self c) Dilemmas around accepting support and adaptations at work	1	5	44 25 63 62 40	20	n.a.	W/Brunel University	UK	Participants tended to measure their recovery in terms of RTW yet continued to experience certain physical and psychosocial difficulties at work related to their illness.
17. Trezzini et al. 2018 [77]	243	Questionnaire a) Work-related outcomes b) Subjective well-being-related consequences	Work-related outcomes: (a) (1) time to RTW; (b) being in paid work at the time of the survey; (c) weekly work time ratio; d) post-SCI work duration ratio. Subjective Well-being-related consequences: (a) household income; (b) quality of life (c) life satisfaction. Covariates: job satisfaction and job type at the time of SCI onset as job-related predictors	3	n.a.	48.26	24.3	53.9	M/ University of Lucerne	Switzerland	45.7% of the participants had returned to their pre-injury employer, 32.9% had changed employers and 21.4% had never returned to paid work post-spinal cord injury (SCI). Returning to the pre-injury employer was associated with a shorter RTW time and a higher current weekly work time compared with starting work with a new employer. No significant differences were found with regard to current employment status and post-SCI work duration.
18. Van der Kemp et al. 2019 [24]	121	Utrecht Scale for Evaluation of Rehabilitation-Participation	Three different aspects of work participation: frequency, restrictions, and job satisfaction	3	1	56.3	27.3	82.6	M7Dutch VSBFonds	The Netherlands	Patients were not in work or were working less than pre-stroke. Ninety percent of those in fulltime employment post-stroke were satisfied with their occupational situation, against 36% of the unemployed participants. Factors predicting RTW, global cognitive functioning and depressive symptoms at two months post-stroke were associated with RTW within one year.
19.Vestling et al. 2003 [75]	120	The subjective well-being scale is one part of *The Göteborg quality of life instrument* Assessment of life satisfaction	Contentment with working environment Subjective Well-being (Physical, Mental and Social) Life Satisfaction	3	1	50	39.17	n.a.	W/ University of Lund	Sweden	Individuals who RTW reported a significantly higher level in subjective well-being and life satisfaction. Being able to walk meant the greatest chance of RTW, followed by white-collar worker, and having preserved cognitive capacity.
20.Yonezawa et al. 2009 [65]	109	Brief job stress questionnaire Short-form 36-item health survey (SF-36) Japanese version The hospital anxiety and depression scale (HADS)	Job stress: job stressors worksite support, level of satisfaction with work or daily life (psychological and subjective well-being) and psychological distress. Quantitative job overload and qualitative job overload represented the psychological job demand. Physical functioning (PF), role-physical (RP), bodily pain (BP), general health perceptions (GH), vitality (VT), social functioning (SF), role emotional (RE), and mental health (MH). Anxiety and depression	2	n.a.	56	17.43	n.a.	H/ Kitasato University	Japan	The Cardiac Rehabilitation group of patients surviving acute myocardial infarction exhibited significantly better results for job stressors and psychological distress and higher health scores at 6 months, as compared with those in the non-Cardiac Rehabilitation group.

Note: n.a.: not available.
